# Supplementary material for: Profiling of Myositis Specific Antibodies and Composite Scores as an Aid in the Differential Diagnosis of Autoimmune Myopathies
Source: Diagnostics (Basel). 2021 Nov 30;11(12):2246. doi: 10.3390/diagnostics11122246 (PMC8699835; doi:10.3390/diagnostics11122246)
Supplement: Supplementary file 1 [file diagnostics-11-02246-s001.zip › diagnostics-1422502-supplementary.pdf]

Table S1. Characteristics of patient cohort

Group	N=	% of IIM	% total cohort
**Myositis**	**264**		
Antisynthetase syndrome (ASS)	67	25.4%	14.4%
Clinically amyopathic dermatomyositis (CADM)	21	8.0%	4.5%
Cancer associated dermatomyositis (cDM)	35	13.3%	7.5%
Dermatomyositis (DM)	70	26.5%	15.1%
Inclusion body myositis (IBM)	6	2.3%	1.3%
Overlap syndrome (OS)	20	7.6%	4.3%
Immune mediated necrotizing myositis (IMNM)	16	6.1%	3.4%
Juvenile dermatomyositis (JDM)	5	1.9%	1.1%
ILD	1	0.4%	0.2%
Polymyositis (PM)	23	8.7%	5.0%
**Controls**	**200**	% of controls	
Rheumatoid arthritis	33	16.5%	7.1%
Systemic lupus erythematosus	40	20.0%	8.6%
Sjögren's syndrome	25	12.5%	5.4%
Infectious disease	40	20.0%	8.6%
Myositis like	20	10.0%	4.3%
Healthy individuals	42	21.0%	9.1%

Table S2. Overview of control samples with at least one positive myositis specific antibody.

		ASS				DM				CADM	IIMN		
Sample ID	Diagnosis	Jo-1	PL-7	PL-12	EJ	Mi-2	NXP2	SAE	TIF1γ	MDA5	HMGCR	SRP	No of pos
NIx0267	SLE	0.1	0.2	0.1	0.2	0.1	0.4	0.1	1.2	0.2	0.1	0.1	1
NIx0281	SLE	0.1	0.2	10.2	0.2	0.1	0.2	0.1	0.1	0.2	0.1	0.1	1
NIx0289	SLE	0.1	0.2	0.1	0.2	0.1	0.2	0.1	0.1	2.1	0.1	0.2	1
NIx0297	SLE	0.1	0.2	0.1	0.2	0.1	2.7	0.1	0.1	0.2	0.2	0.1	1
NIx0302	SLE	0.1	0.3	0.1	0.2	0.1	0.2	0.1	0.1	0.4	2.8	0.2	1
NIx0319	SjS	0.1	0.2	0.1	0.2	0.1	0.4	0.1	0.1	2.2	0.1	0.1	1
NIx0325	SjS	0.1	0.2	0.1	0.2	0.1	2.2	0.1	0.1	0.4	0.1	0.1	1
NIx0360	RA	0.1	0.2	0.1	0.2	0.1	0.3	0.1	0.1	0.2	0.1	7.5	1
NIx0368	RA	0.1	0.2	0.1	0.2	0.1	0.2	0.1	0.1	0.2	0.1	1.6	1
NIx0403	ID	0.1	0.2	0.1	0.3	0.2	0.3	0.2	0.1	0.2	2.8	0.2	1
NIx0409	ID	0.1	0.2	0.1	0.2	0.1	0.2	0.1	0.1	4.1	0.1	0.1	1
NIx0427	HI	0.1	0.2	0.1	0.2	0.1	0.4	0.1	0.1	0.2	0.1	1.2	1
	No of pos	0	0	1	0	0	2	0	1	3	2	2	12

The results derived from PMAT MSA panel for each sample were normalized by dividing the MFI value with respective cutoff value. Values greater than '1' indicate positive result. Abbreviations: ID=infectious disease; HI=healthy individuals; MDA5=Melanoma differentiation-associated protein 5; NXP2=nuclear matrix protein 2; SAE=small ubiquitin-like modifier activating enzyme; RA=rheumatoid arthritis; SLE=systemic lupus erythematosus; SjS=Sjögren`s syndrome; SRP=signal recognition particle; TIF1γ=transcriptional intermediary factor 1 gamma .
